# Host population genetics and biogeography structure the microbiome of the sponge *Cliona delitrix*

**DOI:** 10.1002/ece3.6033

**Published:** 2020-01-24

**Authors:** Cole G. Easson, Andia Chaves-Fonnegra, Robert W. Thacker, Jose V. Lopez

**Affiliations:** ^1^ Department of Biology Middle Tennessee State University Murfreesboro TN; ^2^ Halmos College of Natural Sciences and Oceanography Nova Southeastern University Dania Beach FL; ^3^ Harriet L. Wilkes Honors College Harbor Branch Oceanographic Institute Florida Atlantic University Fort Pierce FL; ^4^ Department of Ecology and Evolution Stony Brook University Stony Brook NY

**Keywords:** *Cliona delitrix*, microbiome, population genetics, porifera

## Abstract

Sponges occur across diverse marine biomes and host internal microbial communities that can provide critical ecological functions. While strong patterns of host specificity have been observed consistently in sponge microbiomes, the precise ecological relationships between hosts and their symbiotic microbial communities remain to be fully delineated. In the current study, we investigate the relative roles of host population genetics and biogeography in structuring the microbial communities hosted by the excavating sponge *Cliona delitrix*. A total of 53 samples, previously used to demarcate the population genetic structure of *C. delitrix,* were selected from two locations in the Caribbean Sea and from eight locations across the reefs of Florida and the Bahamas. Microbial community diversity and composition were measured using Illumina‐based high‐throughput sequencing of the 16S rRNA V4 region and related to host population structure and geographic distribution. Most operational taxonomic units (OTUs) specific to *Cliona delitrix* microbiomes were rare, while other OTUs were shared with congeneric hosts. Across a large regional scale (>1,000 km), geographic distance was associated with considerable variability of the sponge microbiome, suggesting a distance–decay relationship, but little impact over smaller spatial scales (<300 km) was observed. Host population structure had a moderate effect on the structure of these microbial communities, regardless of geographic distance. These results support the interplay between geographic, environmental, and host factors as forces determining the community structure of microbiomes associated with *C. delitrix*. Moreover, these data suggest that the mechanisms of host regulation can be observed at the population genetic scale, prior to the onset of speciation.

## Graphical abstract

We investigated the relative roles of host population genetics and biogeography in structuring the microbial communities hosted by the excavating sponge *Cliona delitrix*. The microbiomes of *Cliona delitrix* were structured by the interplay between geographic, environmental, and host factors. Moreover, these data suggest that the mechanisms of host regulation can be observed at the population genetic scale, prior to the onset of speciation.

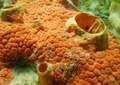

## 1 INTRODUCTION

Marine sponges are an ancient phylum of animals (~600 million years old) that is globally distributed and has successfully colonized a wide range of habitats in shallow and deep seas from tropical to polar latitudes (Bergquist, 1978; Manconi & Pronzato, 2008; Van Soest et al., 2012; Yin et al., 2015). The success of sponges in these systems is linked to their ability to efficiently remove and retain particulate (bacteria, phytoplankton, viruses) and dissolved organic matter from the water column via filter feeding (Maldonado, Ribes, & van Duyl, 2012). Many sponges also host diverse microbial symbiont communities that have likely contributed to their ecological and evolutionary success (Easson & Thacker, 2014; Erwin & Thacker, 2008; Freeman & Thacker, 2011; Lopez, 2019). These internal microbiomes can supplement heterotrophic nutrition from filter feeding by accessing inorganic carbon and nitrogen resources, mediating dissolved organic matter assimilation, and recycling host‐derived nitrogen (Freeman & Thacker, 2011; de Goeij, Berg, Oostveen, Epping, & Duyl, 2008; Southwell, Weisz, Martens, & Lindquist, 2008; Webster & Taylor, 2012).

Early studies of sponge microbiology classified sponge species into two groups based on microbial community abundance: High and low microbial abundance (HMA & LMA, respectively; Hentschel, Usher, & Taylor, 2006; Rützler, 1974; Rützler, 1981; Vacelet & Donadey, 1977) and assigned specific morphological and functional traits to sponges in each group (reliance on microbial symbionts; functional attributes; microbial abundance, diversity, and composition; Weisz, 2006; Weisz, Hentschel, Lindquist, & Martens, 2007). However, recent research eroded this strict dichotomy (Easson & Thacker, 2014; Freeman et al., 2014; Thomas et al., 2016) and indicated a high degree of species specificity in microbial diversity, composition, and function (Freeman, Easson, & Baker, 2014; Reveillaud et al., 2014; Schmitt et al., 2012). In addition, unlike HMA or LMA status (Gloeckner et al., 2014), some of these traits are correlated with host phylogeny (Easson & Thacker, 2014; Freeman et al., 2014; Thomas et al., 2016).

The microbial community composition and functional ecology of sponges might be influenced by local environmental factors, especially in LMA species, since sponges are continuously exposed to a diverse and dynamic consortium of seawater microorganisms via filter feeding. Despite this exposure, most sponge species host microbial assemblages that are distinct from those found in the surrounding seawater (Taylor et al., 2013). In some cases, specific associations are maintained by vertical transfer of microorganisms from parents to eggs and larvae (Diaz, Thacker, Rützler, & Piantoni Dietrich, 2007; Olson & Gao, 2013; Pita, López‐Legentil, & Erwin, 2013; Reveillaud et al., 2014; Schmitt, Weisz, Lindquist, & Hentschel, 2007; Sharp, Eam, Faulkner, & Haygood, 2007).

Across the large biogeographic range of some sponge species, one might expect a highly variable nutritional environment (with varying composition and concentrations of particulate and dissolved organic matter and inorganic nutrients), which might influence host reliance on symbiont‐derived nutrition. However, data from a limited number of species suggest that this reliance is likely species‐specific (Freeman & Thacker, 2011). Some recent studies have demonstrated environmental impacts on sponge microbial communities, with some variation across habitats (i.e., intertidal vs. subtidal, inshore vs. offshore reefs, open water vs. marine lakes) (Cleary et al., 2013; Luter et al., 2015; Weigel & Erwin, 2016), seasons (Hardoim et al., 2012; White et al., 2012), and latitude (Anderson, Northcote, & Page, 2010; Marino, Pawlik, López‐Legentil, & Erwin, 2017; Taylor et al., 2013). Taken together, these studies suggest that sponge‐associated microbial communities are influenced by both host‐specific and environmental factors (Easson & Thacker, 2014; Marino et al., 2017; Taylor, Radax, Steger, & Wagner, 2007; Thomas et al., 2016). At a global scale, microbiomes of individual sponge species exhibited relatively low within‐host‐species variability, suggesting that sponge tissues can form a generally selective habitat at the scale of individual host species; this trend was consistent irrespective of microbial diversity or abundance (Thomas et al., 2016). However, environmental influences on microbial community structure were not explicitly tested in this large‐scale study.

Sponges reproduce by brooding their embryos or through broadcast spawning, in most cases, of fertilized eggs (Maldonado & Bergquist, 2002). Their larval dispersal is limited due to a variety of factors, including planktonic larval duration (<72 hr), transport of eggs and embryo development (<2 weeks), and limited swimming capabilities that leave them at mercy of ocean currents (Chaves‐Fonnegra, Feldheim, Secord, & Lopez, 2015; Maldonado & Riesgo, 2008; Maldonado & Young, 1996). In the Caribbean and western Atlantic, the population structure of sponge species tends to exhibit a high degree of isolation, while connectivity varies in relation to life‐history strategies and the speed of oceanographic currents (Chaves‐Fonnegra et al., 2015; Debiasse, Richards, & Shivji, 2010; Richards, Bernard, Feldheim, & Shivji, 2016). Despite these limiting factors, sponges have been highly successful in their expansion across large latitudinal gradients and diverse environmental conditions in the Caribbean Sea (Van Soest et al., 2012). Possession of robust population genetic data for sponge species spanning large oceanic areas is not yet common (Chaves‐Fonnegra et al., 2015; DeBiasse, Richards, Shivji, & Hellberg, 2016; Swierts et al., 2017), which makes the opportunity to couple this type of data with corresponding bacterial symbiont signals even more compelling. Thus, understanding the intricacies of how these ancient animals can successfully adapt to and colonize divergent environments is still an open question and one of the greater interests under current conditions of global climate change.

**Figure 1:**
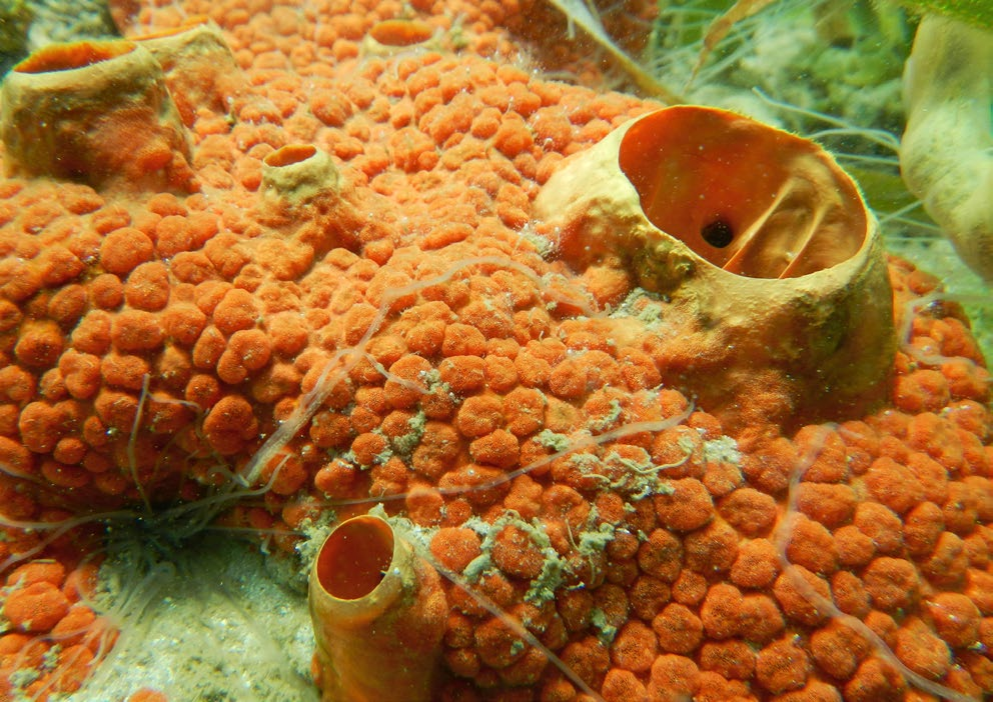
*Cliona delitrix* is an excavating sponge in the family Clionaidae. This species has a wide geographic range that extends from Florida, USA, through the Caribbean, and along the Atlantic coast of South America (World Porifera Database)

As sponges have dispersed and speciated across the globe, it is clear that they have also acquired new microbial symbionts that might have helped them adapt or acclimate to novel nutritional environments (Thomas et al., 2016). While strong patterns of host specificity have been consistently observed, the relationship between host speciation and microbial community composition remains equivocal for many species. Sponge speciation is a continuous process involving slow genetic divergence over time often resulting from reproductive isolation. At intermediate steps of host speciation, potential microbial community divergence might simultaneously occur due to new selective pressures in novel environments, but to date, this question has only been investigated in a few sponge species (Griffiths et al., 2019; Swierts, Cleary, & de Voogd, 2018). Considering that sponges tend to display a high degree of population structure with low dispersal and low connectivity among populations (Chaves‐Fonnegra et al., 2015), comparisons of microbial communities among genetically distinct sponge host populations allow for an evaluation of the potential effects of subspecies host genetic divergence on microbial community structure. In the current study, we coupled sponge population genetics and Illumina‐based microbiome sequencing to investigate the relative influences of host population genetics and biogeography on the microbial communities of the excavating sponge, *Cliona delitrix* (Figure 1). We sampled four distinct populations of *C. delitrix* that were collected at 10 sites across the Caribbean and western Atlantic, with two of the populations having parapatric distributions across Florida and Bahamas reefs. We investigated the microbial taxa that were conserved across this sample set, described their presence and abundance in other sponge species and environments, and tested how geography and population structure are related to microbial community diversity and composition in *C. delitrix*.

## 2 METHODS

### 2.1 Population genetics and geographic distance of host sponges

**Figure 2:**
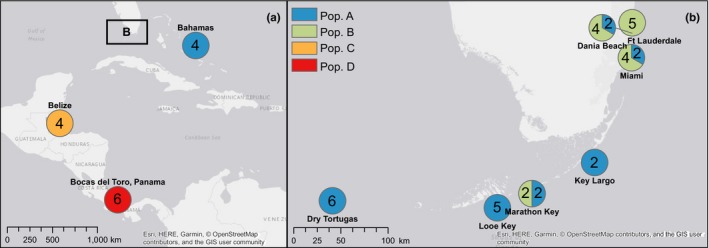
Map of collection locations and population groups. Panel (a) shows the location of Populations C (Belize) and D (Panama) and samples of population A collected in the Bahamas. Panel (b) contains an inset map from panel (a), which shows the collection sites and population composition at each reef along the Florida Reef Tract. Unique population groups as identified in Chaves‐Fonnegra et al. (2015) are displayed as different colors. Numbers within each circle indicate the number of samples collected for each population at each site

A subsample of 53 *C. delitrix* individuals (Figure 1), previously collected and analyzed for population genetic structure by Chaves‐Fonnegra et al. (2015), were chosen to test how geographic distance and population structure are related to microbial community diversity and composition. These sponge samples, which included 4 distinct populations based on 10 microsatellite markers, were selected from 10 locations in the Caribbean Sea and western Atlantic, following a latitudinal gradient from Panama to the eastern Florida Reef Tract and the Bahamas (Figure 2, Table S1).

### 2.2 Statistical design

To analyze and relate population genetic structure and geographic distance among hosts, both discrete and continuous population and geographic variables were included for statistical analyses (Table 1). Discrete population groups were based on population genetic clusters of the host from Chaves‐Fonnegra et al. (2015) and are treated as genetic populations in the present study. Discrete geographic groups were designated at the reef level (site of collection). Continuous genetic distances among populations were calculated as the Bruvo distance, a common method for estimating intraspecific genetic distances among samples based on population genetic data (Bruvo, Michiels, D'souza, & Schulenburg, 2004, Figure 3). Continuous geographic distances were calculated as the Euclidean distance among sampling sites based on the GPS coordinates of each site (Chaves‐Fonnegra et al., 2015).

**Table 1:** Host genetics and geographic factors and variables obtained and used to relate to microbiome diversity and composition

Factors/variables	Discrete	Continuous
Genetic	Genetic population defined in Chaves‐Fonnegra et al. (2015)	Genetic distance
		Bruvo distance from microsatellites
Geographic	Reef—collection site	Geographic distance
		GPS coordinates

**Figure 3:**
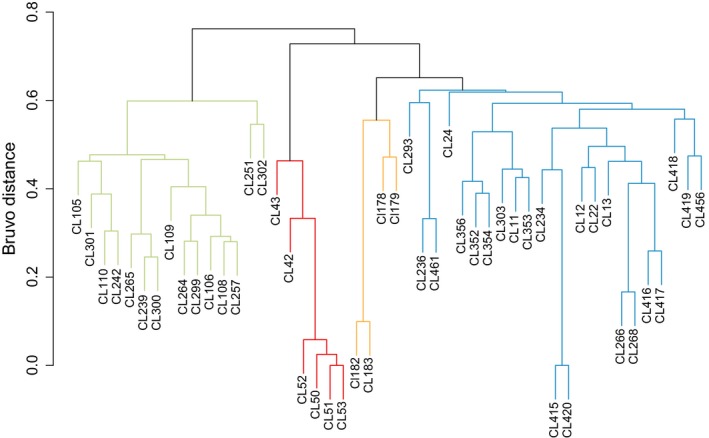
Dendrogram showing the population genetic dissimilarity among C. delitrix samples represented as the Bruvo distance. Unique population groups as identified in Chaves‐Fonnegra et al. (2015) are displayed as different color dendrogram branches. Population A (FL and Bahamas) = blue; population B (FL)  = green; population C (Belize) = orange; population D (Panama) = red

### 2.3 Microbiome analysis

#### 2.3.1 DNA extraction and sequencing

Total genomic DNA was extracted from each sponge sample using the PowerSoil DNA Extraction Kit (MoBio) following the standard protocols of the Earth Microbiome Project (EMP; http://www.earthmicrobiome.org; Thompson et al, 2017). Extracted DNA was shipped to the University of Colorado, Bolder, CO, USA, where the V4 region of the 16S rRNA gene was amplified using the primer set 515F‐806rB and then sequenced on an Illumina HiSeq 2,500 platform (Illumina) following the EMP standard protocols. Sequence processing was performed following the bioinformatics methods outlined in Thomas et al. (2016) and using the program Mothur (Schloss et al., 2009). Sequences were clustered into operational taxonomic units (OTUs), and the identity of each OTU was determined using the SILVA, GreenGenes, and RDP databases (Cole et al., 2013; DeSantis et al., 2006; Quast et al., 2012).

#### 2.3.2 Alpha diversity—all host populations

After converting raw OTU read counts to relative abundance, OTU richness (*S*) and inverse Simpson's diversity (*D*) were compared among population groups and collection sites using an analysis of variance (ANOVA; Oksanen et al., 2016).

#### 2.3.3 Beta diversity—all host populations

Beta diversity (compositional dissimilarity) was calculated among samples using the Bray–Curtis dissimilarity (BCD) calculation. BCD was calculated on two transformations of the OTU data table (relative abundance (RA‐BCD) and presence–absence (PA‐BCD)) to determine the relative dissimilarity associated with changes in microbial taxa abundance and taxa presence. Microbial beta diversity was compared among discrete genetic populations and geographic groups using the permuted multivariate ANOVA (PERMANOVA) function “adonis” in the R package vegan (Oksanen et al., 2016). Pairwise differences among groups were assessed using the pairwise PERMANOVA function in the package RVAidememoire (Hervé & Hervé, 2014), which uses the same PERMANOVA calculations as “adonis” with a multiple comparison correction based on Benjamini and Hochberg (1995). Distance–decay relationships between community composition and the continuous genetic and geographic distances were tested using the Mantel tests and partial Mantel tests (Legendre & Legendre, 2012) implemented in the R package vegan (Oksanen et al., 2016).

#### 2.3.4 Beta diversity—parapatric host populations

Two populations in the current study exhibit parapatry (Chaves‐Fonnegra et al., 2015), while the other two populations were sampled from disparate geographic locations (Belize and Panama). Thus, for these two geographically disparate populations, geographic and genetic effects may be confounding. To better tease apart genetic and geographic effects, we limited some analyses to only the two parapatric population in the seven sites on Florida Reef Tract (Fort Lauderdale, Dania Beach, Miami, Key Largo, Marathon Key, Looe Key, Dry Tortugas) and one site in the Bahamas.

#### 2.3.5 Presence and abundance of taxa across sites and individuals

To assess the conservation of microbial associations across populations and geographic distances, we investigated the presence and abundance of taxa across collection sites and individuals. The occurrence frequency of microbial taxa was assessed across the 10 collection sites to investigate the relationship between occurrence frequency and relative abundance of taxa. We also designated a subset of taxa as “core” taxa. We defined “core” taxa as those OTUs that occurred in at least 42 of the 48 samples (~88%). This cutoff was chosen to allow possible absence of core taxa from geographically disparate samples (i.e., Panama, *n* = 6 or Belize, *n* = 4). After determining the core taxa (OTUs), we queried the presence and abundance of these core taxa in a larger microbiome dataset of sponge and seawater samples (EMP study 1740). This larger dataset was composed of samples that were concurrently sequenced by the EMP and subjected to the same postsequencing processing. This dataset of 1,227 sponge and environmental samples, which includes the samples in the current study, is described in Thomas et al. (2016). OTUs were partitioned into four categories based on their occurrence and abundance in *C. delitrix* and in the larger sponge and environmental samples that were concurrently sequenced by EMP (EMP study 1740; https://qiita.ucsd.edu/emp/study/description/1740), which included three other *Cliona* species. The four categories were as follows: 1) Environmental (found in at least 40% of other EMP samples at similar relative abundances), 2) Sponge‐enriched (detected only in low abundance (<0.1%) in a few (maximum 2% occurrence) in other sponges or environments with higher abundance in *C. delitrix*), 3) *Cliona*‐specific (only detected in *Cliona spp*.), and 4) *C. delitrix*‐specific (unique to *C. delitrix*).

## 3 RESULTS

### 3.1 Host microbiome—final sequences and OTUs

After quality filtering, 22,348 total V4 sequences were obtained from 48 *C. delitrix* samples from 10 distinct sites (Table S1). The raw data for this study are available at the EMP data portal (https://qiita.ucsd.edu/emp/study/description/1740; Study ID: 1740; Thomas, 2014). Before analysis, singletons and OTUs that occurred in fewer than two samples (4%) were removed, which reduced the total number of OTUs to 10,151.

A total of 15 bacterial phyla were detected across *C. delitrix* samples. Individual samples contained a maximum of 15 phyla (CL179—collected in Belize), a minimum of five phyla (CL50—collected in Panama), and a mean (± std. dev.) of 9.21 ± 2.00 phyla per individual sample. The most dominant phylum was Proteobacteria (mean relative abundance ± *SD*: 0.72 ± 0.23), which was largely composed of Gammaproteobacteria. Taxa in the phylum Cyanobacteria were also dominant members of the community, which contained several prokaryotic Cyanobacteria and chloroplast sequences from eukaryotic phytoplankton.

### 3.2 Microbial community in relation to geographic locations and host genetic populations

#### 3.2.1 Alpha diversity

OTU richness (*S*) ranged from 713 to 1991. OTU richness was similar among host genetic populations (ANOVA; *df* = 3, *F* = 1.27, *p* = .30), reefs (ANOVA; *df* = 7, *F* = 0.96, *p* = .47), and the interaction of genetic population and reef (ANOVA; *df* = 2, *F* = 1.15, *p* = .33). Similarly, inverse Simpson's index (*D*) relationships were similar among genetic populations (ANOVA; *df* = 3, *F* = 1.65, *p* = .20), reefs (ANOVA; *df* = 7, *F* = 0.54, *p* = .80), and the interaction of host population and reef (ANOVA; *df* = 2, *F* = 0.32, *p* = .73).

#### 3.2.2 Beta diversity—discrete—geographic and genetic groups

We observed significant differences in RA‐BCD associated with all discrete factors when each was considered separately (PERMANOVA; genetic population: *p* = .001; reef: *p* = .001; interaction: *p* = .05). However, significant overlap among independent variables was observed when all factors were considered in the same PERMANOVA (Table 2). We did observe a significant interaction between reef and genetic population (PERMANOVA; *p* = .05), and together, these two factors accounted for nearly half of the variance among samples (Table 2). These results may suggest overlap in the explanatory power of these factors at this spatial scale. Pairwise PERMANOVA revealed significant differences among all genetic populations, but differences among reefs were not always significant (Table S2). For PA‐BCD, we observed that a significant portion of the variance associated with each factor was associated with the presence of unique taxa (RA‐R^2^/PA‐R^2^; genetic population: 0.24/0.19; reef: 0.40/0.34).

**Table 2:** Statistical results for microbiome diversity and composition analysis of all sites and populations

Dataset/factors	Statistical test	*F*	*df*	*R* ^2^	*p*
*Community richness*					
Host population	2‐way ANOVA	1.27	3		.3
Reef		0.96	7		.47
Host population × Reef		1.15	2		.33
*Inverse Simpson's diversity*					
Host population	2‐way ANOVA	1.65	3		.20
Reef		0.54	7		.80
Host population × Reef		0.32	2		.73
*Beta diversity—discrete—geographic and genetic groups*					
Host population	PERMANOVA	4.65	3	0.24	**.001**
Reef		2.89	9	0.41	**.001**
Host population × Reef		1.55	2	0.05	**.05**
*Beta diversity—continuous—geographic and genetic gradients*				***r***	***p***
Geographic distance	Mantel test			.42	**.001**
Genetic distance	Mantel test			.31	**.001**
Geographic | Genetic	Partial mantel test			.35	**.001**
Genetic | Geographic	Partial mantel test			.18	**.001**

**Figure 4:**
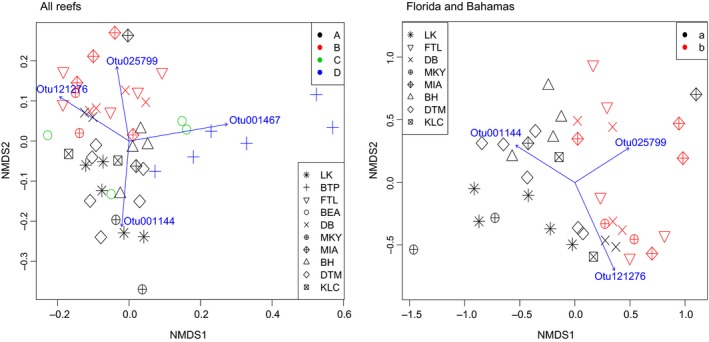
NMDS plots of microbial community dissimilarity among samples from all sites (left panel) and Florida and Bahamas sites (right panel). Blue vectors and text indicate trends in the relative abundance of specific OTUs that were drivers of significant differences among population groups. Three OTUs were the main drivers in both sample sets including OTU001144 (Gammaproteobacteria), OTU121276 (Gammaproteobacteria), and OTU025799 (Stramenopiles), which distinguished population A from population B (Florida and Bahamas populations), while OTU001467 (Stramenopiles) separated population D (Panama population) from the other population groups

Further examination of the dataset revealed that approximately 54% of all observed OTUs (*S*) occurred at two or fewer reefs. Simper analysis revealed several OTUs that were the primary drivers of BCD differences among genetic populations (Figure 4). These OTUs include several taxa belonging to Gammaproteobacteria and some eukaryotic taxa (detected via chloroplast sequences) in Stramenopiles (diatoms) and Ulvophyceae (green algae).

Samples collected in Panama had a high abundance of a Bacillariophyta (Diatom) taxon (Otu001467; detected via chloroplast sequence; mean relative abundance = ~36%) compared to other samples (<1% mean relative abundance). While this taxon accounted for a large portion of the differences between Panama and other populations/locations (Simper; ~22% of total variance), it did not occur broadly enough in *C. delitrix* samples to be considered a core taxon. Within the broader concurrently sequenced EMP dataset (study 1740), this taxon occurred in 526 samples outside the current dataset and was abundant in several mangrove species (*Tedania ignis, Haliclona tubifera, and Dysidea etheria*) that were also collected in Bocas del Toro, Panama, in close proximity to the *C. delitrix* individuals that were sampled for this study. Diatoms have been previously documented as sponge symbionts (Sipkema & Blanch, 2010; Taylor, Schupp, Dahllöf, Kjelleberg, & Steinberg, 2004), mostly in polar regions, but the broad occurrence of this taxon in other species, especially those collected nearby, suggests that this taxon is perhaps more likely to be a food source whose abundance is somewhat reflective of the water column in this region of Panama. Belize samples were distinct from other population groups and sites largely by the higher relative abundance of two taxa. Otu001969 was a Gammaproteobacteria core taxon that was particularly abundant in samples from Belize (mean relative abundance = ~9%) compared to others (mean relative abundance = ~0.02%, 0.04%, and 3.7% for populations A, B, and D, respectively). Otu000623 was found in 21 samples and classified as a member of Ulvophyceae, which is a family mostly comprised of green macroalgae. Our data in the current study are not sufficient to determine whether this alga and sponge have a symbiotic relationship as has been found in other studies (Carballo & Ávila, 2004; Davy, Trautman, Borowitzka, & Hinde, 2002; Easson, Slattery, Baker, & Gochfeld, 2014; Pile, Grant, Hinde, & Borowitzka, 2003; Trautman, Hinde, & Borowitzka, 2000), or simply an environmental contaminant acquired during the collection of this excavating sponge species.

#### 3.2.3 Beta diversity—continuous—geographic and genetic gradients

We observed significant correlation between RA‐BCD and both geographic (Mantel test; *r* = 0.42, *p* = .001) and genetic (Bruvo) distances (Mantel test; *r* = 0.31, *p* = .001). The partial Mantel tests revealed that both population and geographic distances remained significant even when the variance of each distance was initially removed (partial Mantel test; geographic distance | genetic distance: *r* = 0.35, *p* = .001; genetic distance | geographic distance: *r* = 0.18, *p* = .001). These partial Mantel tests revealed that geographic distance was more strongly correlated with RA‐BCD than genetic distance; however, the effect of genetic distance on beta diversity remained significant in the partial Mantel test.

#### 3.2.4 Beta diversity—parapatric populations Florida Reef Tract and Bahamas

We observed significant differences in RA‐BCD among genetic population groups and reefs, even when only considering parapatric populations. These two factors exhibited a significant overlap in variance (~8%) and a significant interaction (PERMANOVA; Genetic Population: *p* = .001; Reef: *p* = .001; Genetic population × Reef: *p* = .044, Table 3, Figure 4). Mantel tests showed a lack of a spatial distance–decay relationship among samples (Mantel test; *r* = 0.08, *p* = .16), but a significant correlation with genetic distance (Mantel test; *r* = 0.17, *p* = .001). Similar to previous results, a partial Mantel test showed that genetic distance remained significantly correlated after geographic distance effects were first removed (partial Mantel; genetic distance | geographic distance: *r* = 0.16, *p* = .002; Table 3). These results indicate that while a significant reef effect remains, the distance–decay relationship is lost when the most geographically disparate sites (Belize and Panama) are excluded. Additionally, the contrasting results of the PERMANOVA and Mantel tests suggest that geographic distance likely influences sponge microbiomes at the smaller scale of individual reefs. Thus, these effects are not likely due to large‐scale spatial gradients within the greater Caribbean region.

**Table 3:** Statistical results for microbiome composition analysis of parapatric populations along the Florida Reef Tract and Bahamas

Dataset/factors	Statistical test	*F*	*df*	*R* ^2^	*p*
*Beta diversity—discrete geographic and genetic groups*					
Host population	PERMANOVA	6.2	1	.12	**.001**
Reef		2.02	7	.28	**.001**
Host population × Reef		1.7	2	.07	**.04**
*Beta diversity—continuous—geographic and genetic gradients*				*r*	*p*
Geographic distance	Mantel test			.08	.16
Genetic distance	Mantel test			.17	**.001**
Genetic | Geographic	Partial Mantel test			0.16	**0.002**

#### 3.2.5 Presence and abundance of taxa across sites and individuals

**Figure 5:**
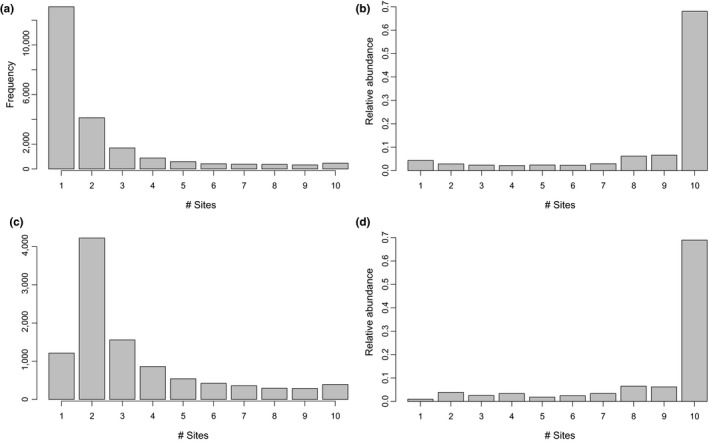
OTU occurrence frequency and relative abundance across 10 collection sites for raw (A & B) and cleaned datasets (C &D). A & C show the number of OTUs that are present in 1–10 sites (*x*‐axis) in the current study. B & D display the relative abundance of OTUs that occur in the occurrence frequency bins shown in A & C

Initially, most taxa found in the 48 *C. delitrix* samples occurred in a single individual (Figure 5), which is observed by comparing OTU presence and frequency in a single reef before (Figure 5a) and after (Figure 5c) a data cleaning step that specifically removed OTUs present in fewer than two samples. While the majority of OTUs were found at two or fewer reefs, the most abundant members of the *C. delitrix* community were found at all reefs. Of the 391 OTUs found at all reefs, 63 of them were labeled as core OTUs because they were present in at least 42 of 48 individual samples. Four of the core OTUs, all of which were in the phylum Cyanobacteria, were classified as “Environmental” since they were found in a broad array of EMP samples (from EMP study 1740) at similar abundances to *C. delitrix* samples (0.1%–4%). Ten OTUs were “Sponge‐enriched,” showing sporadic occurrence (max = 18 occurrences) and lower relative abundance in other EMP samples (in EMP study 1740) compared to *C. delitrix* samples in this study. An additional eleven OTUs were “*Cliona‐*specific” and occurred in low abundance in six *Cliona celata* samples collected from the southern coast of Portugal. These same OTUs were not detected in a second congener, *Cliona viridis*, which was collected at the same location as *C. celata*. Lastly, 38 OTUs were “*C. delitrix*‐specific,” and surprisingly, these unique OTUs were all low abundance taxa (mean ± *SD*: 0.03 ± 0.01% of total community).

#### 3.2.6 Sponge‐enriched OTUs

**Figure 6:**
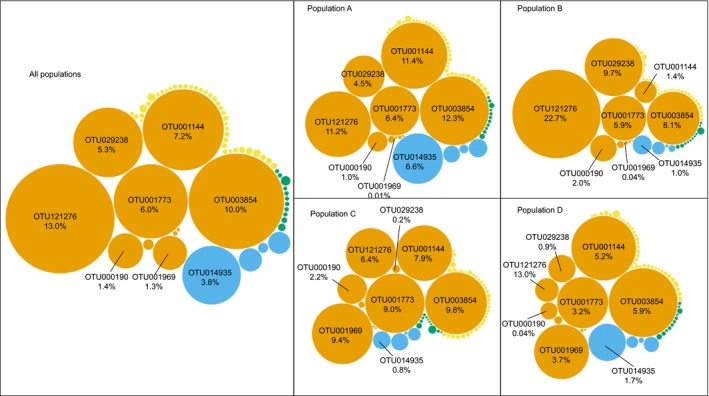
Bubble plot of 63 core taxa in *C. delitrix* samples showing variability in the mean relative abundance in core OTUs among populations of *C. delitrix*. Each plot represents the same 63 core taxa, and the eight most abundant taxa (in all populations) are labeled with their respective OTU identities in each panel. Each bubble represents one core OTU, and bubble size indicates the relative abundance of an OTU. Core taxa categories are represented as different colors: Blue—Environmental; orange—Sponge‐enriched; green—*Cliona*‐specific; yellow—*Cliona delitrix*‐specific

**Figure 7:**
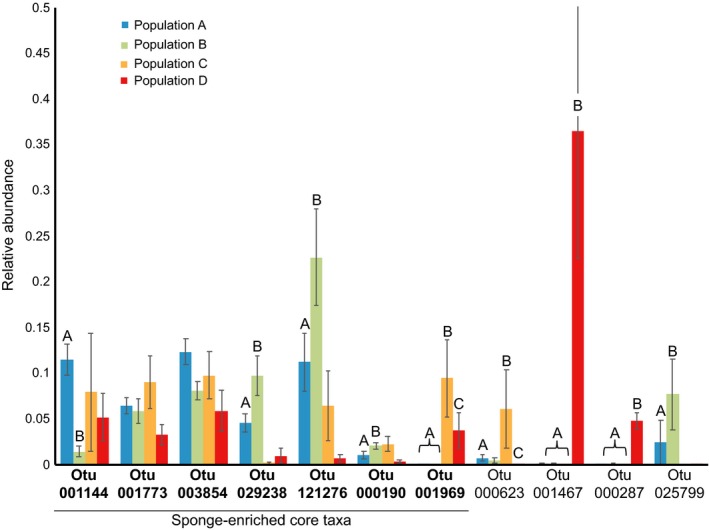
Relative abundance of 11 OTUs (7 sponge‐enriched core taxa) that account for significant differences among population groups (Simper analysis). The letters above the bars (A, B, C) indicate statistically significant differences among population groups; (a) FL‐Bahamas population group A, (b) FL‐Bahamas population group B, (C) Belize population group, (D) Panama population groups. The seven bolded taxa along the x‐axis represent sponge‐enriched core taxa, and all are classified as Gammaproteobacteria except for Otu003854, which is only classified as a Proteobacteria taxon. The remaining four taxa were not members of the core taxa group and are classified as Ulvophyceae (Otu000623), Stramenopiles (Otu001467), Gammaproteobacteria (Otu000287), and Stramenopiles (Otu025799)

The ten sponge‐enriched OTUs, all of which were Proteobacteria, comprised 3%‐70% (mean ± *SD*: 43 ± 18%) of the total microbial community in *C. delitrix*. Samples collected from Panama (population D) had the lowest mean abundance for the enriched taxa (mean ± *SD*: 19 ± 16%). However, the Belize samples (population C; mean ± *SD*: 43 ± 10%) and the two populations in Florida (population A: mean ± *SD*: 45 ± 15%; population B: mean ± *SD*: 49 ± 18%) both showed higher mean relative abundances for these ten enriched OTUs (Figure 6). When we compared enriched OTU relative abundance to genetic and geographic distance variables, we found a significant independent (Mantel test; genetic dist.: *r* = 0.24, *p* = .001; spatial dist.: *r* = 0.37, *p* = .001) and partial effect of both variables (partial Mantel test; genetic dist. | geographic dist.: *r* = 0.12, *p* = .006; geographic dist. | genetic dist.: *r* = 0.31, *p* = .001). These results indicate that both host genetics and geographic distance are factors likely important in structuring the sponge‐enriched taxa in the broader *C. delitrix* microbiome (Figure 7).

At the individual OTU level, we observed these mixed effects with the factors genetic population and reef both showing significant effects on the relative abundance of sponge‐enriched OTUs (ANOVA; Tukey's HSD; Table 4). Specifically, seven OTUs showed significant differences, one with significant variation among reef, five with significant differences among genetic populations, and one with significant differences related to both reef and genetic population (Table 4).

**Table 4:** Sponge‐enriched core OTUs. Mean relative abundance, standard deviation, and taxonomic classification of sponge‐enriched OTUs. Significant effects and pairwise differences show which independent variables showed significant differences among groups

OTU	Mean rel. abundance	Standard deviation	Lowest tax classification	Significant effects	Pairwise differences
Otu001144	0.069	0.078	Gammaproteobacteria	Site & Pop	Pop A > B
Otu001773	0.058	0.044	Gammaproteobacteria	NS	
Otu003854	0.096	0.059	Proteobacteria	NS	
Otu029238	0.052	0.064	Gammaproteobacteria	Pop	Pop B > A, C, D
Otu121276	0.127	0.166	Gammaproteobacteria	Pop	Pop B > D
Otu000190	0.013	0.015	Gammaproteobacteria	Pop	
Otu000491	1.20E‐03	1.28E‐03	Gammaproteobacteria	Site	
Otu001969	0.012	0.036	Gammaproteobacteria	Pop	Pop C > A, B, D; Pop D > A, B
Otu003852	2.34E‐04	1.79E‐04	Proteobacteria	Pop	
Otu007558	1.20E‐04	9.70E‐05	Proteobacteria	NS	

#### 3.2.7 Genus‐ and species‐specific OTUs

The majority of the core OTUs were specific to the genus *Cliona* or the species *C. delitrix* when compared to a concurrently sequenced dataset of 1,227 sponge and environmental microbiomes. However, all of these OTUs were low abundance taxa and the maximum abundance of these taxa in any individual was 0.7% (Otu001869). Eleven of these low abundance OTUs appear to be conserved across some members of the genus *Cliona*, specifically *Cliona celata*. *C. delitrix* and *C. celata* were collected from opposite sides of the Atlantic and processed at separate institutions before being sent to EMP (Thomas et al., 2016). Additionally, other sponge species in this broader dataset (study 1740) were collected at the same sites as these two species, yet these rare, genus‐specific OTUs were not detected in their microbiomes.

## 4 DISCUSSION

The present study focuses on the symbiotic microbiomes of *C. delitrix* and represents one of the first integrative studies to link host and microbial genetics on a broad scale. Variation in the symbiotic microbial community associated with *C. delitrix* was related to both host population genetics (genetic group; Chaves‐Fonnegra et al., 2015) and biogeography. Our analysis revealed a significant relationship between geographic distance (i.e., a distance–decay relationship) and change in microbial community composition, but only across large geographic distances (~15° latitude). Significant microbiome variation was not observed over smaller geographic distances (e.g., within the Florida Reef Tract), which is likely due to the high observed variability at the level of individual collection reef. Significant, but moderate, correlations between microbiome variation and host population genetics were observed at all geographic scales. This finding suggests that host specificity in sponge microbiomes may be more complex than previously documented and perhaps sensitive to even small, population‐level genetic variation. Although considerable variation was observed among samples, several taxa were conserved across geographic and genetic groups. A total of 63 core OTUs were designated as core taxa. Ten of these core taxa were “sponge‐enriched,” and these taxa were consistently the most dominant members of *C. delitrix* microbiomes. Forty‐nine core taxa were specific to the genus *Cliona* or to *C. delitrix* samples when compared to a concurrently sequenced dataset of over 1,200 sponge and environmental microbiomes. Surprisingly, these genus and species‐specific taxa occurred at low abundance in all samples despite their persistent presence.

The interplay between host and environmental forces is a common theme in microbiome research. Some research in sponge microbiology suggested that high environmental influence and low host specificity would occur in sponges that host a low abundance of microbes (i.e., LMA species; e.g., Webster et al., 2013). However, recent studies have observed a strong and persistent effect of host species irrespective of geography, microbial abundance, or microbial community complexity. Several studies that have observed a relationship between microbial community structure and environmental variability have also noted strong host effects (e.g., Cleary et al., 2013; Griffiths et al., 2019; Hardoim et al., 2012; Marino et al., 2017; Reveillaud et al., 2014), with most of the environmental variability being observed within species. A recent study on geographic variation in the microbiome of the sponge *Ircinia campana* observed significant variation in microbial communities only over large geographic gradients, but also noted the presence of location‐specific microbial taxa (Marino et al., 2017). Similarly, *C. delitrix* microbial communities in the current study exhibited significant intraspecific variation in microbial community composition at large latitudinal gradients (~15°), while small‐scale geographic effects were largely due to site‐specific microbial taxa. Large‐scale geographic gradients in the current study were mostly driven by differences among samples from Belize and Panama, when compared to the eight other collection reefs that contained the two remaining genetic population groups.

Population genetics of the host (based on microsatellites loci) showed clear differentiation among genetic clusters from the Caribbean (Panama and Belize) and Atlantic (Florida and Bahamas; Chaves‐Fonnegra et al., 2015), and this pattern was mirrored by the differentiation of symbiotic microbial communities. Along with these spatial gradients and site differences is a persistent signal of the host related to both discrete genetic group (Chaves‐Fonnegra et al., 2015) and dissimilarity in genetic variation at microsatellite loci. This observation was particularly striking when we narrowed the geographic extent of sampling to only include the two parapatric populations along the Florida Reef Tract and Bahamas. In this instance, spatial effects (distance–decay) were absent, but a moderate correlation with genetic dissimilarity remained. Previous studies have documented a connection between host genetics and associated microbial communities and shown that community structure (i.e., alpha diversity) exhibits a phylogenetic signal whereby more closely related species have more similar community diversity (Easson & Thacker, 2014; Schöttner et al., 2013; Thomas et al., 2016). However, these studies have also suggested that selection for divergent microbial community composition remains strong even among closely related species; thus, selection for specific membership in these microbiomes appears to be regulated mostly at the species level (Easson & Thacker, 2014). Our findings in this study support these previous results through both a lack of within‐species differences in alpha diversity and a correlation with host genetic variation and microbial community composition. These results might indicate that as these hosts begin to genetically diverge from one another, possibly due to reproductive isolation, the composition of their associated microbial communities will begin to reflect it, even when the divergence is minimal.

### 4.1 Core communities

The concept of a “core” symbiont community (taxa that are shared among individual hosts) was initially used to highlight taxa that were shared across many host species collected from different locations. For sponges, this hypothesis was proposed to support the occurrence of a uniform microbial community among diverse sponge species (Hentschel et al., 2002). As research on sponge microbiomes has progressed, the term “core” has varied greatly in its definition and use (Astudillo‐García et al., 2017; Schmitt et al., 2012). In the current study, we used it to describe symbiont taxa that occurred in at least 88% of collected samples. With the increasing sequencing depth afforded by next‐generation sequencing techniques, we now understand that many “core” symbiont taxa are detectable in the environment and thus likely enriched from it (i.e., horizontally acquired; Taylor et al., 2013). Ten OTUs in the current study were both core and sporadically detected in other sponge hosts or environmental samples in the sponge microbiome project (Easson & Thacker, 2014; Thomas et al., 2016). Eight of these OTUs composed the dominant members of most *C. delitrix* symbiont communities, with the exception of samples collected from Panama population (Figure 6). While these eight core members comprised 45%–54% of the community in populations A‐C (Florida, Bahamas, and Belize), in population D (Panama) samples (*n* = 6), these same OTUs only represented approximately 20% of the community. It is perhaps surprising that the most dominant members of *C. delitrix* microbiomes are potentially acquired from the environment, rather than vertically transmitted. If these taxa are horizontally transmitted, *C. delitrix* juveniles must have a mechanism for selecting these specific taxa out of the vast number that exist in the environment similar to those mechanisms in other symbiotic systems (Baker et al., 2019; Mcfall‐Ngai, 2014; Nyholm & McFall‐Ngai, 2004).

The majority of the core OTUs were specific to either the genus *Cliona* or *C. delitrix*. These OTUs, however, all occurred at values of low relative abundance, with a maximum relative abundance of 0.7% in a single sample. The observed low relative abundance is most likely below the detection limit of many earlier methods for assessing symbiont communities (e.g., clone libraries). Low abundance of these taxa could be interpreted as indicating that these taxa play relatively minor ecological roles in the holobiont, but the persistence of these rare taxa in hosts separated by wide geographic expanses and evolutionary time suggests otherwise. Some recent research has focused on microbial taxa found at low abundance, terming them the “rare biosphere” and explored the forces driving the dynamics of the rare biosphere. In some systems, rare taxa are disproportionately active and can exhibit widely varying temporal profiles in abundance and activity (Sogin et al., 2006). Such wide temporal variation is often indicative of an environmental response to seasonal changes or disturbances (Lynch & Neufeld, 2015). Although the current study did not sample over time, the spatial sampling scheme, which covered much of the species range of *C. delitrix*, may be somewhat analogous, as environmental variability would be expected over both season and the spatial extent of this study. Environmental conditions in locations such as Bocas del Toro Panama, an embayment with high allochthonous inputs of nutrients (Aronson, Hilbun, Bianchi, Filley, & Mckee, 2014), would be expected to be quite different than those in the Bahamas or on offshore reefs in the Florida Keys. Despite these potential environmental influences, the relative abundance of genus and species‐specific taxa remained remarkably consistent across our samples, which may suggest an important role for these rare low abundance bacteria.

Much of the research into the rare biosphere has focused on free‐living systems (e.g., bacterioplankton), but in a symbiotic system, constraints on microbial abundance could be quite different due to host–symbiont interactions. The abundance of rare taxa could also be driven by top‐down forces such as phage predation or host immune responses, which require taxa to remain rare to avoid detection (reviewed in Lynch & Neufeld, 2015). If this scenario explains the rare taxon abundance in the current study, it might signal that at least some of these taxa are parasitic cheaters that have found a way to persist in association with the host sponge. Rare taxa can also represent a genetic “seed bank” of ecological potential by containing a diverse cache of metabolic machinery. In the context of the current study, this scenario would suggest that these rare taxa are important in specialized situations, such as other life stages (e.g., larval stages) or at time points crucial to the species (e.g., during spawning). An environmental or simply stochastic explanation for the presence of these rare taxa seems implausible given their persistence across the Caribbean in *C. delitrix* and in some cases across the Atlantic Ocean in *C. celata*, as well as their conspicuous absence in other host taxa (even sympatric species), but additional research is needed to adequately address this hypothesis. Sequencing the microbiomes of *C. delitrix* larva and adults during spawning would likely provide additional evidence as to the role of these rare but persistent taxa.

Our study explored variability in sponge microbial communities associated with variation in sponge microbiomes and, similar to previous studies, found an interplay between host and geographic forces in structuring the microbiome of *C. delitrix*. Variability among *C. delitrix* microbiomes was apparent at large geographic scales, while a significant but moderate host population genetic correlation was observed independent of geography. Although variation among samples was observed, several bacterial taxa were consistently found in *C. delitrix* samples. The most dominant core taxa were also observed at low abundance in the environment, while the *Cliona*‐specific taxa all occurred at low abundance within the host sponge. To date, microbiome divergence has been observed at the level of host species with limited evidence of divergence among populations (Griffiths et al., 2019; Swierts et al., 2018). The results of the current study add to this body of evidence and indicate that microbiome divergence can be observed at the population genetic scale, prior to the onset of speciation.

## AUTHOR CONTRIBUTIONS

A. Chaves‐Fonnegra designed the study and prepared the samples for sequencing. C.G. Easson and R.W. Thacker processed the sequence data, designed and implemented the data analysis, and wrote the first draft of the manuscript. C.G. Easson, A. Chaves‐Fonnegra, J.V. Lopez, and R.W. Thacker contributed to the writing and editing of the manuscript.

## Data Availability

All sequence data used in this study are available through the Qiita server at (https://qiita.ucsd.edu/emp/ , Study ID –1740 (https://qiita.ucsd.edu/emp/study/description/1740; Thomas, 2014).
